# Genetic damage biomarkers in buccal epithelial cells of healthy individuals staying near three mobile phone base stations

**DOI:** 10.1186/1755-8166-7-S1-P49

**Published:** 2014-01-21

**Authors:** Naresh Mahajan, Akbar Bhat, Gursatej Gandhi

**Affiliations:** 1 Department of Human Genetics, Guru Nanak Dev University, Amritsar, India

**Keywords:** Radiation, gentic damage, micronucli

## Background

Massive surge in mobile phone usage throughout the world has increased the installation of mobile phone base stations to meet the subscribers demand. However, as mobile phone towers and handset use microwave radiation for signal transmission, their arise health concerns from exposures to these radiations especially for people living in areas where the mobile phone base stations have been installed.

## Materials and methods

In cross sectional case control study, effort was made to assess genetic damage in some individuals (n=50; exposed group) residing in an area with three base stations close by (150 meters) with power density ranging from 172.6 to 550.3µw/m^2^ at the threshold of residences from where sampling was done. Healthy controls (n=50) with no exposure were contacted from areas with no base stations (power density 0.0023µw/m^2^).The standard buccal cytome assay was performed to assess DNA (genetic) damage, all proliferation and cell death biomarkers.

## Results and Conclusion

Genetic damage (micronuclei and nuclear buds) was significantly (p<0.05) higher in the exposed group while cell proliferation markers (basal cells and binucleated cells) were not significantly raised. However, among the cell death markers (condensed chromatin, karyorrhectic cells, pyknotic cells and karyolitic cells), the condensed chromatin (p<0.05) and karyolitic (P<0.05) cells also showed significant increase in the exposed group. The observed significant increase in genetic damage assessed in the buccal epithelial cells in individuals residing near mobile phone base stations is of concern as all neoplasia initiate from genetic damaging events.

